# Cholesterol and Lipoprotein Dynamics in a Hibernating Mammal

**DOI:** 10.1371/journal.pone.0029111

**Published:** 2011-12-15

**Authors:** Jessica P. Otis, Daisy Sahoo, Victor A. Drover, Chi-Liang Eric Yen, Hannah V. Carey

**Affiliations:** 1 Department of Comparative Biosciences, University of Wisconsin, Madison, Wisconsin, United States of America; 2 Department of Medicine, Medical College of Wisconsin, Milwaukee, Wisconsin, United States of America; 3 Department of Nutritional Sciences, University of Wisconsin, Madison, Wisconsin, United States of America; The University of Queensland, Australia

## Abstract

Hibernating mammals cease feeding during the winter and rely primarily on stored lipids to fuel alternating periods of torpor and arousal. How hibernators manage large fluxes of lipids and sterols over the annual hibernation cycle is poorly understood. The aim of this study was to investigate lipid and cholesterol transport and storage in ground squirrels studied in spring, summer, and several hibernation states. Cholesterol levels in total plasma, HDL and LDL particles were elevated in hibernators compared with spring or summer squirrels. Hibernation increased plasma apolipoprotein A-I expression and HDL particle size. Expression of cholesterol 7 alpha-hydroxylase was 13-fold lower in hibernators than in active season squirrels. Plasma triglycerides were reduced by fasting in spring but not summer squirrels. In hibernators plasma β-hydroxybutyrate was elevated during torpor whereas triglycerides were low relative to normothermic states. We conclude that the switch to a lipid-based metabolism during winter, coupled with reduced capacity to excrete cholesterol creates a closed system in which efficient use of lipoproteins is essential for survival.

## Introduction

**Figure 1:**
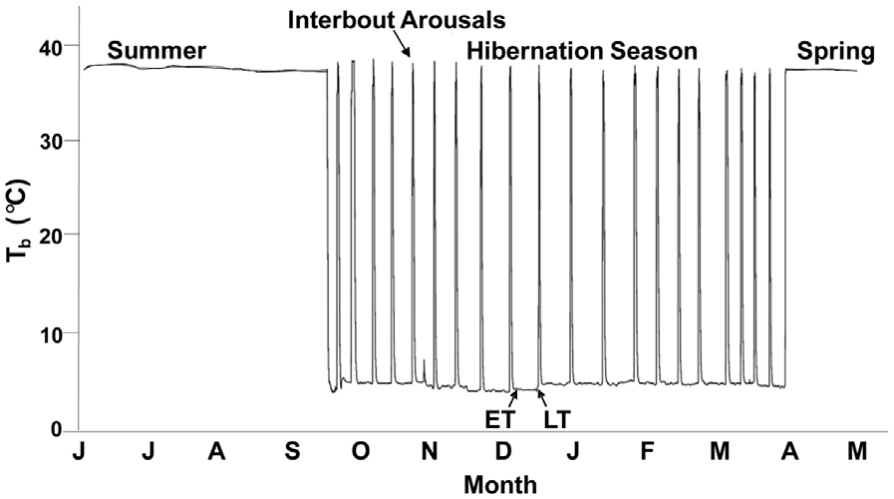
Body temperature (T_b_) changes over the annual cycle of the 13-lined ground squirrel. The T_b_ trace during hibernation was obtained with a telemeter implanted in a squirrel housed in an environmental chamber maintained at 3–4°C. T_b_ lines in spring and summer are hand-drawn for illustration. Winter torpor bouts last from a few days to 2–3 wk, and interbout arousals (IBA) to normothermia typically last <24 h. ET, early torpor; LT, late torpor.

Hibernation is a seasonal adaptation that facilitates survival during harsh environmental conditions [1], [2]. Hibernating ground squirrels fast during the winter months, spending much of the time in a depressed metabolic state known as torpor which provides substantial energy savings. Torpor bouts last from a few days to several weeks and are interrupted by periodic arousals to normothermia (Fig. 1). Energy demands during winter are met primarily by oxidation of fatty acids liberated from white adipose tissue (WAT). During the active season hibernators accumulate large fat depots such that body mass can double from spring to early fall [3]. It is well established that lipids play crucial roles in hibernation biology through their contribution to energy metabolism during the winter fast and their effects on membrane composition, which have been linked to torpor patterns [3], [4], [5], [6], [7]. Less well understood is the effect of hibernation on lipid trafficking, especially cholesterol and lipoprotein dynamics, over the annual cycle and its functional significance [8], [9], [10], [11].

Cholesterol can be obtained from the diet or synthesized *de novo* by any cell in the body. Cholesterol and lipids are transported in the circulation by lipoprotein particles: chylomicrons transport gut-derive triglycerides (TGs); very low density lipoprotein (VLDL) and low density lipoprotein (LDL) particles distribute liver-derived TGs and cholesterol to peripheral tissues; and high density (HDL) particles transport excess cholesterol from the periphery to the liver for excretion [12] . Excess cholesterol is lost from the body via fecal excretion or by conversion to bile acids. There is great interest in understanding the function and regulation of HDL cholesterol (HDL-C) and apolipoprotein A-I (apoA-I), the main structural component of HDL particles, because they are associated with protection from cardiovascular disease.

Previous work demonstrated that apoA-I mRNA and protein expression in the liver and intestine, the two organs that synthesize apoA-I, increases during hibernation in ground squirrels [13], [14], [15]. This is consistent with elevations in plasma cholesterol that have been observed in ground squirrels and other species during hibernation [8], [9], [16], [17]. However, the mechanisms responsible for changes in plasma cholesterol and apoA-I expression in hibernation are not well understood, and a comprehensive analysis of cholesterol and lipoprotein dynamics over the annual hibernation cycle in a single species has not been conducted. In this study we used the 13-lined ground squirrel (*Ictidomys tridecemlineatus*) to examine several steps in cholesterol transport and storage in plasma and tissues shortly after termination of hibernation, in late summer during the fattening phase and in various hibernation states of torpor and arousal. Our investigation focused on circulating cholesterol and on key cholesterol-handling organs: liver, intestine, and WAT. Our results highlight the dynamic nature of lipid and sterol handling in a mammal that undergoes extreme shifts in food intake each year and relies on massive fat deposition for survival.

## Methods

### Animals

All procedures were approved by the University of Wisconsin-Madison Institutional Animal Care and Use Committee (protocol numbers V1134, V1339 and V1383) and follow the guidelines set by the Public Health Service Policy on Humane Care and Use of Laboratory Animals. Thirteen-lined ground squirrels were collected in the vicinity of Madison, WI in May, July, August and September. Experimental groups included squirrels born in captivity to pregnant females collected in May, and adult (≥1 yr) and juvenile (<1 yr) male and female squirrels collected in July, August and September. During the spring and summer, squirrels were housed at 22°C with a 12L∶12D cycle. Wild-caught squirrels were provided with water and rat chow (Purina 7001, St. Louis, MO) *ad libitum* supplemented with sunflower seeds to provide n-6 poly-unsaturated fatty acids required for normal hibernation patterns. Squirrels born in captivity were restricted to 12 g of rat chow/d beginning at weaning to prevent excessive gain in body mass. In July, a subset of squirrels were implanted with VitalView Series 3000 wireless telemeters (Philips Respironics, Bend, Oregon) or with iButtons temperature data loggers (Dallas Semiconductor, Dallas, TX) for body temperature (T_b_) monitoring. Telemeters were calibrated by submerging each unit in water baths set to low (4°C) and high (40°C) temperatures to mimic expected T_b_ ranges. Frequencies produced at the two temperatures were entered into Vital View software to convert telemeter frequencies recorded *in vivo* to actual T_b_s. The iButtons were pre-calibrated by the manufacturer over a wide temperature range (−40 to +80°C) with ±1°C accuracy. Beginning in September, squirrels were transferred to a room maintained at 4°C with constant darkness, except for brief (∼5 min) periods of dim light each day to check activity states using the sawdust method [18]. Food and water were removed once squirrels began regular torpor bouts. All squirrels showed regular torpor-arousal cycles for ≥3 wk before euthanasia.

Active squirrels (T_b_ ∼37°C), were studied in spring (SPR) or summer (SUM) in either the fed or fasted (18–20 h) states. SPR squirrels were sampled in May, 1 mo after terminating hibernation in captivity; SUM squirrels were sampled in July or August. During hibernation, squirrels were sampled during entrance into torpor (EN, T_b_ 20–25°C), early torpor (ET, within 24 h of reaching minimum T_b_ ∼5°C), late torpor (LT, ≥7 d in torpor, T_b_∼5°C), arousing from torpor (AR, T_b_ 20–25°C), and in natural interbout arousal (IBA, T_b_ 37°C) (Fig. 1). Squirrels were euthanized by isoflurane anesthesia (Baxter Healthcare Corporation, Deerfield, IL) followed by decapitation, except for ET and LT animals which did not receive isoflurane due to their extremely low respiratory and metabolic rates. Tissues and plasma were frozen in liquid nitrogen and stored at −80°C until analysis.

### Time course of plasma cholesterol changes

Six squirrels not used in other experiments were subjected to repeat blood draws at (i) 4 wk and 2 wk prior to entering the cold room; (ii) on the day of cold room entry, and (iii) at 1, 2 and 3 mo after starting hibernation. Prior to blood draw, hibernators were transferred to a warm, lighted room for 4 h to induce an arousal. When rectal temperature was ≥35°C, blood was drawn and squirrels were then returned to the cold room. To draw blood, all squirrels were anesthetized with an isoflurane/O_2_ mixture (3%/L) and the pedal vein was pierced with a 23 gauge needle. Blood (∼100 µl) was collected in heparinized micro-hematocrit capillary tubes and stored on ice. Plasma was collected by centrifugation and stored at −80°C until analysis.

### Plasma cholesterol, lipid, and β-hydroxybutyrate analysis

Plasma cholesterol was measured with Infinity Cholesterol Liquid Stable Reagent (Thermo Scientific, Asheville, NC). Free cholesterol (FC) in the plasma was quantified with the Wako Free Cholesterol E kit (Wako Diagnostics, Richmond, VA). Esterified plasma cholesterol was calculated by subtracting FC from the total cholesterol in each sample. Plasma TGs were measured with Infinity Triglycerides Reagent (Thermo Scientific, Asheville, NC). All assays were performed in duplicate according to the manufacturer's instructions.

Plasma lipoprotein profiles were determined for SUM fasted (n = 6), SUM fed (n = 5), SPR fasted (n = 5), LT (n = 5) and IBA (n = 6) squirrels following the methods in Miyazaki, MYC et al. [19]. Briefly, 100 µl of plasma was diluted 1∶1 with PBS and filtered with a Cameo 3AS syringe filter (0.22 Mm). The samples were injected onto a Superose 6HR 10/30 fast protein liquid chromatography column (Amersham Pharmacia Biotech, Piscataway, NJ) equilibrated with PBS containing 1 mM EDTA and 0.02% NaN_3_, with a constant flow rate of 0.3 ml/min. We measured total, free and esterified cholesterol, as well as TGs in each fraction with the reagents outlined above, reporting values as total mass/fraction.

β-hydroxybutyrate (BHB) was measured in fresh plasma with the LiquiColor kit (StanBio Laboratory, Boerne, TX) according to the manufacturer's instructions (n = 5 or 6 for all groups).

### Tissue lipid and sterol analyses

To measure total body cholesterol, SUM Fasted (n = 3), SPR Fasted (n = 3), and LT (n = 6) squirrels were euthanized and the plasma, intestine, liver, brain, and remaining carcass were collected, weighed and stored at −80°C. Plasma cholesterol concentration was measured as described above.

The carcass was homogenized in a blender with liquid nitrogen until powdered; tissues were powdered on dry ice. A portion of each homogenate was subjected to Folch extraction. Briefly, powdered tissue was combined with 2/1 chloroform/methanol in a 1∶20 ratio, agitated for 10 min, filtered (Watman #1 filter paper), 37% KCl was added, and the samples were spun at 3,000×*g* for 10 min. The organic phase was dried under nitrogen. Cholesterol content was measured by adding 1 ml of working reagent (15 ml phenol reagent (80.8 mM phenol in 50 mM PIPES buffer, pH 6.9), 15 ml mixed reagent (2 mM 4-aminoantipyrine, 6 mM sodium cholate, 200 mM potassium chloride, and 0.2% Triton-X in 50 mM PIPES buffer pH 6.9), 7.5 U cholesterol oxidase, 7.5 U cholesterol esterase, and 375 U peroxidase) to each sample, mixing, incubating at 37°C for 20 min, cooling to room temp, measuring absorbance at 500 nm, and calculating cholesterol content in mg by comparison to a standard curve [20]. Total cholesterol content of each organ and the carcass were determined and whole body cholesterol was calculated for each animal by summing total plasma, carcass, liver, intestinal and brain cholesterol.

TG, free fatty acid (FFA), FC, and cholesteryl ester (CE) content of liver, WAT and intestinal mucosa (n = 5 or 6 for all) from a different subset of animals were measured by high-performance thin layer chromatography (HP-TLC) as described previously [21], [22]. Resulting bands were quantified by densitometry and expressed relative to a standard.

### Biliary constituents

After euthanasia, liver and gallbladder were exposed. Gallbladder bile was collected by inserting a syringe into the gallbladder and aspirating its entire contents. Bile was stored at −80°C. Cholesterol, phospholipid, and bile acid concentrations were measured for SUM Fed (n = 7) and LT (n = 6) squirrels in the laboratory of Dr. Stephen Turley (University of Texas Southwestern) using methods described previously [23].

### qRT-PCR

qRT-PCR was performed as previously described [24]. Briefly, RNA was isolated and cDNA synthesized from 1 µg of RNA. qRT-PCR was performed with Power SYBR Green qPCR Master Mix (Invitrogen, Carlsbad, CA) containing ROX dye using an Applied Biosystems StepOnePlus machine. Primers were synthesized by Integrated DNA Technologies and the sequences are listed in Table S1. We used combinations of reference genes that have stable expression in the gut and liver of 13-lined ground squirrels over the annual hibernation cycle; the genes used were acidic ribosomal phosphoprotein, succinate dehydrogenase complex subunit A, and hypoxanthine-guanine phosphoribosyltransferase for intestine, and cyclophilin A, glyceraldehyde 3-phosphate dehydrogenase, and tyrosine 3-monooxygenase for liver [24].

### Immunoblotting

SDS-PAGE was performed on plasma (1 µl), and liver cytosolic fractions. Equal protein loading was confirmed by Ponceau S staining for plasma and by normalization to β-actin expression for liver samples, which does not vary over the annual hibernation cycle [25]. Protein bands were quantified with ImageJ software (NIH, Bethesda, MD). Antibodies used in this study were raised against apoA-I (Rockland #600-101-109, Gilbertsville, PA), cholesterol 7 alpha-hydroxylase (CYP7A1) (gift of Dr. Steven Russell, University of Texas Southwestern), and β-actin (Cell Signaling #4967, Danvers, MA).

### MGAT activity

Liver monoacylglycerol acyltransferase (MGAT) activity was measured as previously described [26]. All assays were performed at 37°C and activity was expressed as pmol of acyl CoA incorporated into diacylglycerol (DG) and TG per min per mg protein in the liver homogenate.

### Data analysis

Results are presented as means ± s.e.m. and significance was defined as P<0.05. Comparisons of two groups were made by t-test. Comparisons of three or more groups were made by one-way ANOVA followed by Fisher's post-hoc test. To determine VLDL-C, LDL-C and HDL-C, the FPLC fractions corresponding to each lipoprotein class were summed. Plasma cholesterol and body mass changes over time were analyzed with one-way repeated-measure ANOVAs followed by t-tests with Bonferroni corrections.

## Results

### Plasma cholesterol

**Figure 2:**
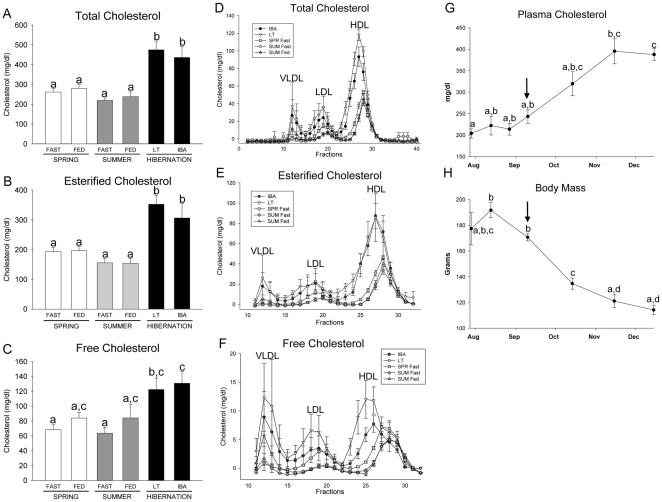
Plasma and lipoprotein cholesterol concentrations over the annual cycle. Plasma total, esterified, and free cholesterol (A–C) in fasted and fed spring (SPR) (open bars) and summer (SUM) (grey bars) squirrels, and in hibernators (filled bars) during late torpor (LT), and interbout arousal (IBA) are shown. Total, esterified, and free cholesterol of fast protein liquid chromatography fractionated lipoproteins from a separate set of squirrels are shown (D–F). Plasma cholesterol (G) and body mass (H) of a third set of fed squirrels at 6, 4, and 2 wk prior to hibernation, on the day they entered the cold room (indicated with arrow), and at 4, 8 and 12 wk of hibernation are also shown. Groups with the same letter are not different. All values represent means ± s.e.m., n = 5 or 6.

Total plasma cholesterol levels were similar in spring and summer squirrels and were not affected by an overnight fast (Fig. 2A; body masses and temporal information for the experimental groups are presented in Tables S2 and S3). Cholesterol levels in hibernators were nearly 2-fold higher than in active squirrels (Fig. 2A). Plasma cholesterol was largely esterified (Figs. 2B,C) and, similar to other rodents, the majority was carried in HDL particles (Fig. 2D). HDL-C and LDL-C levels were greater in LT and IBA compared with SPR and SUM squirrels (Fig. 2D). There was a similar trend for increased VLDL-C in LT and IBA squirrels compared with active squirrels (P = 0.388). Interestingly, HDL particles were larger in LT and IBA hibernators than in SPR and SUM squirrels, as indicated by a chromatographic shift to the left of the HDL-C peak (Figs. 2B–F). Consistent with increased HDL-C, plasma apoA-I protein was greater in hibernators (ET, LT and IBA combined; 246.7±6.2 arbitrary units, n = 15) than in active season squirrels (SPR and SUM combined; 185.6±10.9 arbitrary units, n = 10; P<0.0001).

To investigate temporal changes in plasma cholesterol during hibernation we sampled plasma from 6 animals for 2 mo prior to hibernation and for 3 mo during hibernation. Plasma cholesterol was stable during late summer and early fall (∼225 mg/dl), but gradually increased after 2 and 3 mo of hibernation (∼375 mg/dl) (Fig. 2G). Body masses of these squirrels were greatest in late summer and early fall and gradually fell during the sampling period, reaching their lowest winter values when plasma cholesterol was highest (Fig. 2H).

### Tissue cholesterol

Tissue cholesterol (normalized to wet tissue mass) was measured in active squirrels after an overnight fast to minimize variability due to recent food intake. Whole body cholesterol content was similar in SPR, SUM and LT squirrels; however, because body mass was lower in LT than SPR or SUM absolute cholesterol levels were lower in LT hibernators (Table 1). Cholesterol content was greater in brains of LT than in SPR or SUM squirrels, but was similar among the groups for liver and intestine (Table 1).

**Table 1:** Whole body and tissue cholesterol levels in ground squirrels.

	Spring Fast	Summer Fast	Late Torpor
**Whole body**	12.64±1.06	9.82±0.49	10.78±0.46
**Liver**	23.99±7.88	21.52±1.08	18.16±0.65
**Intestine**	12.96±0.55	15.86±0.50	17.53±1.4
**Brain**	6.19±3.58	5.69±3.29	7.22±2.95*
**Plasma**	293.85±31.97	250.17±33.79	511.21±33.90*

Values are means ± s.e.m. n = 3 for spring and summer; n = 6 for late torpor,

*indicates late torpor is different from spring and summer in the same row. Cholesterol units are mg/g body mass for whole body, mg/g tissue mass for organs and mg/dl for plasma.

**Figure 3:**
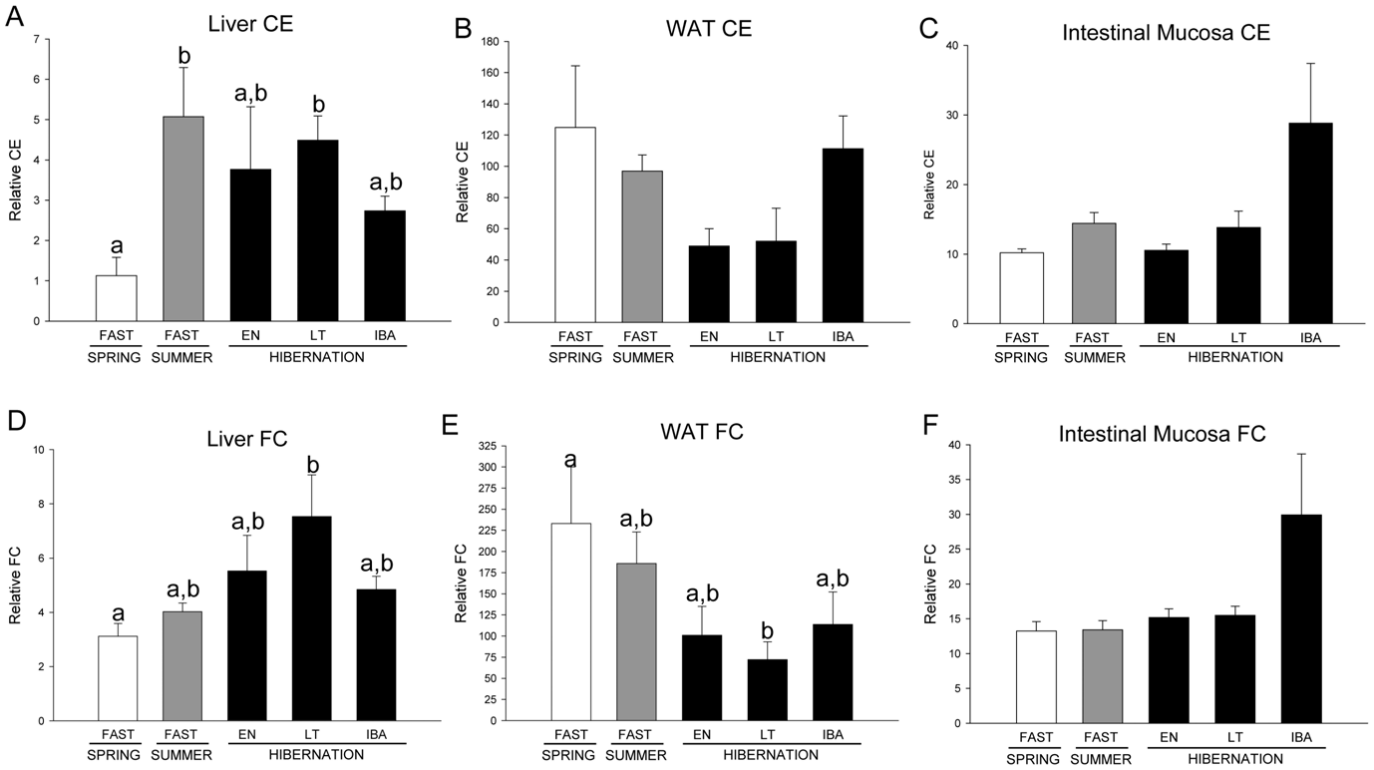
Tissue cholesterol levels in ground squirrels. Liver, white adipose tissue (WAT), and intestinal mucosa cholesteryl ester (CE) (A–C) and free cholesterol (FC) (D–E) levels in fasted spring (SPR) (open bars) and summer (SUM) (grey bars) squirrels, and in hibernators (filled bars) in entrance (EN), late torpor (LT), and interbout arousal (IBA) are shown. Groups with the same letter are not different. All values represent means ± s.e.m., n = 5 or 6 for all groups.

Liver CE normalized to tissue mass was greater in SUM and LT compared with SPR squirrels (Fig. 3A). FC level in livers of LT squirrels was greater than in SPR (Fig. 3D). The opposite pattern was found in WAT, as FC level was higher in SPR than in LT (Fig. 3E). Despite trends for higher levels in IBA hibernators, CE and FC in intestinal mucosa did not vary among the groups (Fig. 3C, F).

### Expression of lipid- and cholesterol-related genes

**Figure 4:**
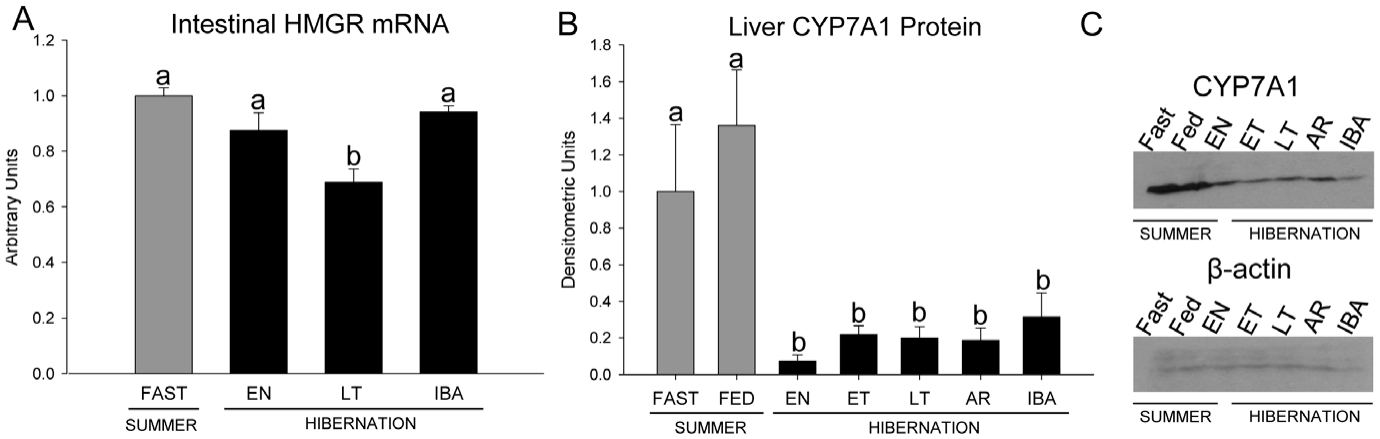
HMG-CoA reductase (HMGR) intestinal mRNA expression and Cholesterol 7 alpha-hydroxylase (CYP7A1) liver protein expression. Gene and protein expression in fasted summer (SUM) (grey bars) squirrels and hibernators (filled bars) in entrance (EN), early torpor (ET), late torpor (LT), arousal (AR), and interbout arousal (IBA) are shown. HMGR gene expression is normalized to reference genes (acidic ribosomal phosphoprotein, succinate dehydrogenase complex subunit A, and hypoxanthine-guanine phosphoribosyltransferase) and expressed relative to SUM which was set at 1.0. CYP7A1 expression is normalized to β-actin protein expression (example blot panel C). Groups with the same letter are not different. All values represent means ± s.e.m. and n = 5 or 6.

We measured mRNA expression of several genes related to lipid and cholesterol metabolism in intestine and liver of fasted SUM squirrels, and in EN, LT and IBA hibernators. Intestinal mRNA expression was similar among the groups for the ATP-binding cassette transporter A1 (ABCA1), Neimann-Pick C1 like 1 (NPC1L1), apoA-IV, ATP-binding cassette sub-family G member 8 (ABCG8), liver X receptor (LXR), and microsomal triglyceride transfer protein (MTTP) (data not shown). However, expression of HMG-CoA reductase (HMGR) mRNA was lower in intestine of LT compared with SUM and EN squirrels (Fig. 4A). In liver, mRNA expression was similar among the groups for ABCA1, LXR, sterol regulatory element binding protein (SREBP) 1c, SREBP2, HMGR, LDL receptor (LDLR), and acyl-CoA∶cholesterol acyltransferase (ACAT1) (data not shown).

### Bile constituents

Cholesterol concentration and molar ratio in gallbladder bile was greater in LT relative to fed SUM squirrels (Table 2). Biliary phospholipids were also greater in LT (Table 2). In contrast, biliary bile acid concentration was similar in SUM and LT squirrels and the molar ratio of bile acids was lower in LT (Table 2). Liver protein expression of CYP7A1, the rate limiting enzyme in the conversion of cholesterol to bile acids, was reduced ∼13-fold in hibernation compared with SUM squirrels (Fig. 4B).

**Table 2:** Biliary lipid composition in summer and late torpor ground squirrels.

	Summer Fed	Late Torpor
**Cholesterol (µM/ml)**	8.26±1.09	16.72±1.84*
**Cholesterol (%)**	3.2±0.2	4.8±0.05*
**Bile Acids (µM/ml)**	207.2±21.5	256.9±10.2
**Bile Acids (%)**	79.7±1.0	74.1±1.2*
**Phospholipids (µM/ml)**	43.6±4.0	73.9±6.9*
**Phospholipids (%)**	17.1±1.0	21.1±1.1*

Concentrations and molar ratios (%) of cholesterol, bile acids and phospholipids from summer fed (n = 7) and late torpor (n = 6) squirrels. Values are means ± s.e.m.,

*indicates late torpor is different from summer fed in the same column.

### Plasma and tissue TGs

**Figure 5:**
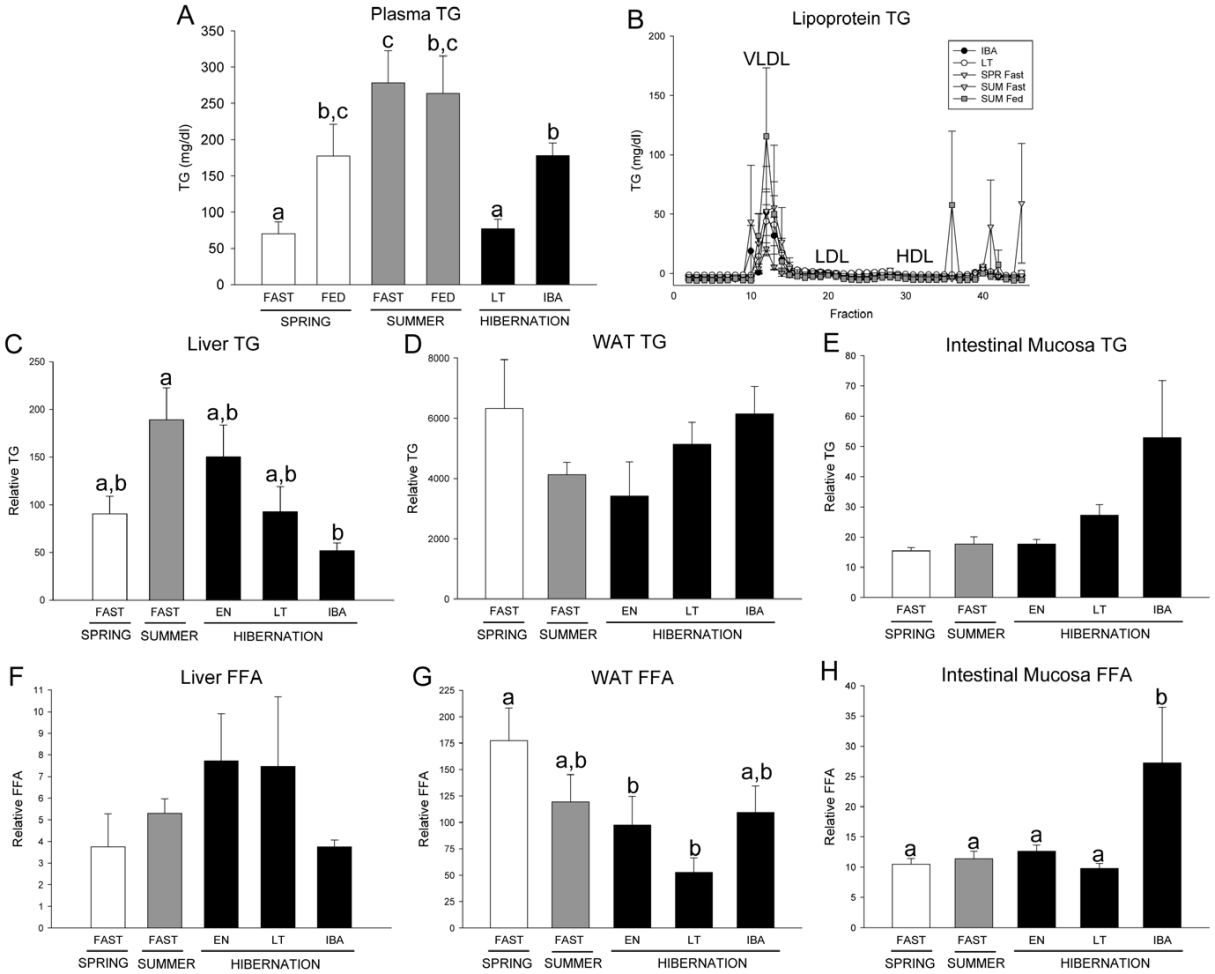
Plasma, lipoprotein, and tissue triglycerides (TG) and free fatty acids (FFA) over the annual hibernation cycle. (A) Total plasma and (B) lipoprotein TGs following fractionation by FPLC in fasted and fed spring (SPR) (open bars) and summer (SUM) (grey bars) squirrels, and in hibernators (filled bars) during late torpor (LT), and interbout arousal (IBA) squirrels are shown. Tissue TG (C, D, E) and FFA levels (F, G, H) in liver, white adipose tissue (WAT) and intestinal mucosa in fasted SPR, SUM, entrance (EN), LT and IBA squirrels are shown. Groups with the same letter are not different. All values represent means ± s.e.m., n = 5–7.

Plasma TGs were reduced by fasting in SPR but not SUM squirrels, and although levels in SPR and SUM squirrels were similar under fed conditions, plasma TGs were greater in SUM in the fasted state (Fig. 5A). In the hibernation states examined, plasma TGs were lower in LT compared with IBA and both were lower than in SUM (Fig. 5A). As expected, lipoprotein TG analysis revealed that plasma TGs were located in VLDL particles (Fig. 5B).

We measured TG and FFA levels in the liver, WAT and intestinal mucosa in active squirrels under fasted conditions and in hibernators during EN, LT and IBA. In liver, TG levels normalized to tissue mass differed only between SUM and IBA squirrels (Fig. 5C). Concentrations of TGs in WAT were similar among the groups (Fig. 5D). There were no differences in FFA content in liver, but FFA levels in WAT were higher in SPR than in EN and LT squirrels (Fig. 5G). As observed for CE and FC, intestinal mucosa TG levels tended to be highest in IBA hibernators (Fig. 5E) and mucosal FFA concentration was significantly higher in IBA squirrels compared with the other groups (Fig. 5H).

### MGAT activity and Plasma BHB

**Figure 6:**
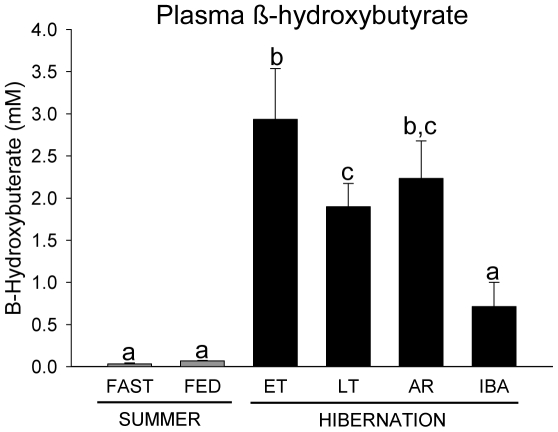
Plasma β-hydroxybutyrate (BHB) concentration over the annual hibernation cycle. BHB concentrations in fasted and fed spring (SPR) (open bars) and summer (SUM) (grey bars) squirrels, and in hibernators (filled bars) during early torpor (ET), late torpor (LT),arousing (AR), and interbout arousal (IBA) squirrels are shown. Groups with the same letter are not different. All values represent means ± s.e.m., n = 5 or 6 for all groups.

There were no differences in MGAT activity among SPR Fast (1685.31±125.76 pmol/min/mg), SPR Fed (1.49±0.11 nmol/min/mg), LT (1.39±0.12 nmol/min/mg), and IBA squirrels (1.28±0.13 nmol/min/mg) (n = 5 for all groups). Plasma BHB levels were greater in ET, LT and AR squirrels compared with SPR, SUM, and IBA groups (Fig. 6).

## Discussion

**Figure 7:**
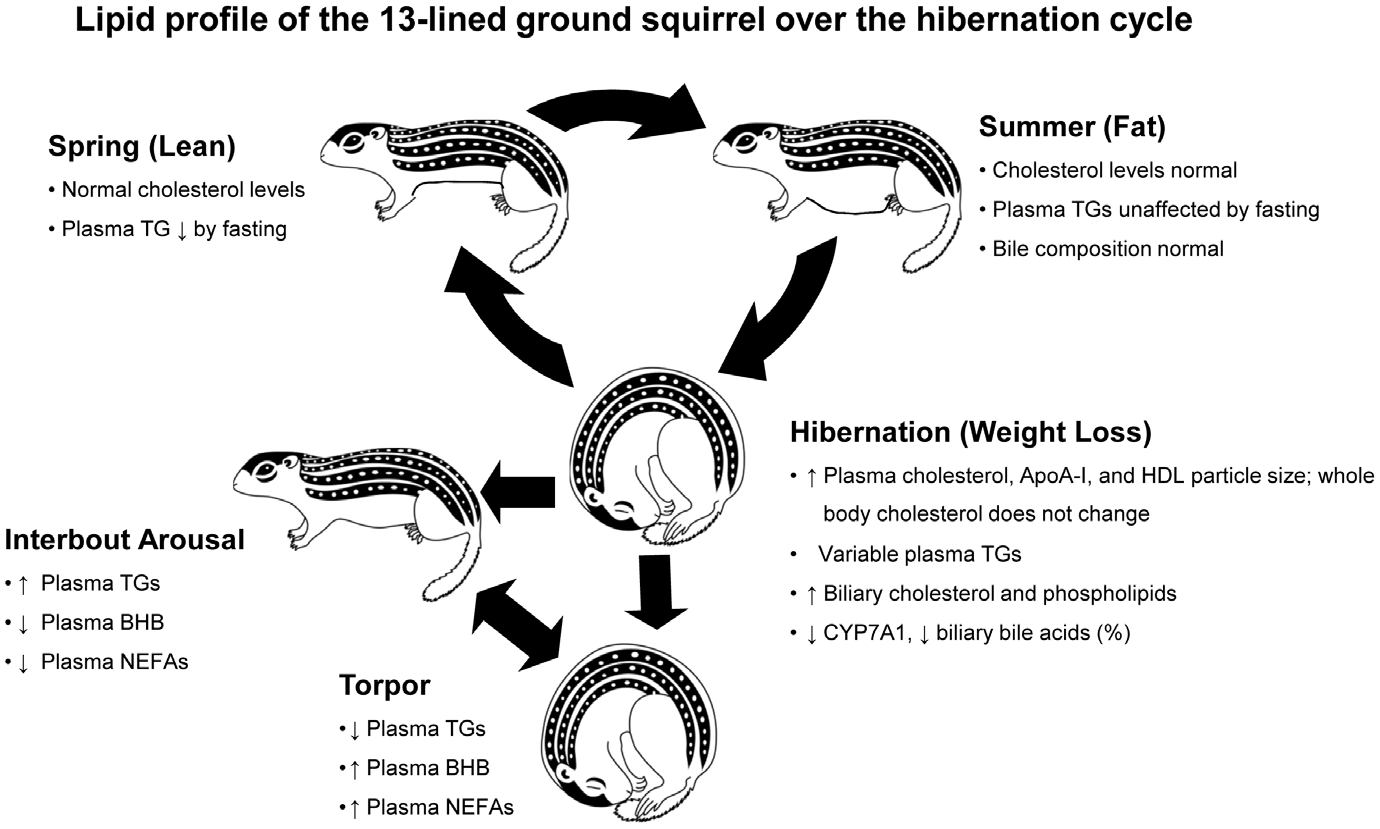
Schema of the metabolic profile of the 13-lined ground squirrel over the annual hibernation cycle. Abbreviations: TG, triglyceride, ApoA-I, apolipoproteinA-I, LT, late torpor, ET, early torpor, IBA, interbout arousal, CYP7A1, cholesterol 7 alpha-hydroxylase; BHB, β-hydroxybutyrate.

In this study we exploited the hibernation model of seasonal mass gain and long-term fasting to explore cholesterol and lipoprotein dynamics in the face of extreme nutritional change, the findings of which are summarized in Fig. 7. Each year, seasonal hibernators undergo major shifts in nutrient intake and fuel utilization that result in a switch to a primarily lipid-based metabolism during the winter months; As noted in other species [8], [9], [16], [17], [27], hibernation increased plasma cholesterol levels in 13-lined ground squirrels. Plasma cholesterol concentration was similar in SPR and SUM squirrels, but rose gradually over the first two months of hibernation concomitant with a decrease in body mass. These changes were accompanied by increases in plasma HDL-C and LDL-C, as well as apoA-I, the major protein component in HDL particles. A novel finding of our study was the increase in HDL particle size in hibernators, consistent with a greater amount of cholesterol carried within each particle. This change is intriguing because very small and very large HDL particles are associated with increased risk of atherosclerosis [28], [29], [30]. In mice, the absence of scavenger receptor BI (SR-BI), the HDL receptor, also increases HDL particle size [31]. Although attempts to measure SR-BI levels in squirrels were unsuccessful, further investigation into mechanisms responsible for the seasonal change in HDL size may shed light on regulation of HDL particle structure in pathologic states. To better understand the basis for the hibernation-induced increases in plasma cholesterol, we measured total cholesterol, CE and FC levels in key cholesterol handling organs. Despite the dramatic effect of hibernation on circulating levels, total cholesterol concentrations in whole body, liver and intestine were similar in hibernating squirrels and fasted, active squirrels. This differs from a previous metabolomic study of 13-lined ground squirrels that revealed higher total liver cholesterol levels in torpid vs. summer squirrels, although the latter group was not fasted [32].

The effect of hibernation on plasma cholesterol levels resembles that of fasting in non-hibernating mammals. Fasts of a few days to several weeks increase plasma total cholesterol, HDL-C and lipoprotein levels in rodents and rabbits [33], [34], [35], [36], [37]. Fasting also increases plasma cholesterol levels in humans [38], [39], [40]. Plasma cholesterol concentration is a function of the balance between processes that affect cholesterol input into and excretion from the body, all of which can be affected by hibernation. Cholesterol input occurs via dietary absorption by the enterocyte NPC1L1 transporter; cholesterol then enters the plasma in nascent HDL particles and chylomicrons [12]. Cholesterol can also be synthesized by all tissues with the majority occurring in liver and intestine [41]. The winter fast eliminates dietary cholesterol intake in hibernators, making *de novo* synthesis the only way that new cholesterol is added to body stores. Although we did not measure rates of cholesterol synthesis in squirrel tissues, it is likely reduced relative to the active season. Fasting in other rodents reduces hepatic and intestinal cholesterol synthesis [41], [42], and we detected no changes in HMGR mRNA in hibernation. Because cholesterol synthesis requires energy expenditure, its reduction during hibernation would contribute to energy conservation. Yet, a basal level of cholesterol synthesis must continue during the winter fast because biliary cholesterol concentrations are maintained (and even increase, likely due to biliary fluid reabsorption in intervals between gallbladder emptying). Biliary cholesterol levels also rise in hibernating bears [10], but are unchanged in hibernating golden-mantled ground squirrels [43]. Although some cholesterol secreted into bile in hibernators is excreted during interbout arousals, some may be reabsorbed via the intestinal epithelium via NPC1L1. Our results indicate that NPC1L1 mRNA is expressed in ground squirrel intestine. Intestinal cholesterol absorption via NPC1L1 during interbout arousals may help to minimize the need for *de novo* cholesterol synthesis, which is energetically costly.

Cholesterol levels in plasma can also be augmented by efflux of free cholesterol to circulating HDL particles [12]. This might be significant for hibernators as WAT depots are gradually depleted to provide fatty acids for oxidation. As TGs are hydrolyzed and adipocytes shrink, cellular cholesterol pools would be effluxed to plasma and contribute to the rise in plasma cholesterol.

In addition to fecal losses, cholesterol is excreted via its conversion to bile acids. Expression of CYP7A1 the rate-limiting enzyme in bile acid synthesis was reduced 13-fold in livers of hibernating squirrels. A reduction (2.8-fold) in CYP7A1 mRNA was also observed in hibernating black bears [44]. These results suggest that reduced synthesis of bile acids contributes to the rise in plasma cholesterol during hibernation. We found relatively few differences in mRNA levels of other genes involved in cholesterol homeostasis in intestine and liver of fasted SUM squirrels and hibernators. This is remarkable given that a 20 h fast in mice severely reduces expression of liver HMGR, SREBP1c, SREBP2 and LDLR [34]. One explanation is that the 20 h fast we imposed in SUM squirrels already reduced mRNA expression to levels that could not be reduced further by the longer winter fast.

Taken together, our results suggest that plasma cholesterol rises during hibernation as a consequence of seasonal fattening followed by winter fasting. As hibernation proceeds, cholesterol pools are effluxed into plasma as large WAT stores are depleted, conversion of cholesterol to bile acids is reduced via downregulation of CYP7A1, and the lack of food intake reduces the capacity for fecal cholesterol excretion. This altered dynamic is well tolerated by the animals, and in fact there is evidence that elevated plasma cholesterol may play a functional role in the hibernation phenotype. Provision of a high cholesterol diet prior to hibernation in chipmunks increased plasma cholesterol levels and was associated with longer torpor bouts and a lower minimal body during torpor [45], both of which contribute to energy conservation. In addition, the increased expression of apoA-I by liver and intestine during hibernation, which is consistent with the greater need to transport circulating cholesterol in HDL particles, may benefit hibernators due to the additional functions that have been attributed to this apolipoprotein. ApoA-I has anti-inflammatory and antioxidant properties [46], [47], and HDL particles have been linked to innate immunity [48]. Hibernation is associated with greater resistance to ischemia-reperfusion injury including suppression of inflammatory responses [49], [50]. Elevated cholesterol levels in hibernators may also facilitate the synthesis of sex hormones that occurs shortly after hibernation terminates, coincident with the start of the breeding season [51].

Intestinal CE, FC, TG and FFA concentrations all tended to be higher during interbout arousals compared with active season squirrels or with other hibernation states. These observations suggest that despite its dissociation from nutrient processing and the substantial atrophy during hibernation [52], the intestine may contribute to metabolic processes during periodic arousals. Increased expression of intestinal apoA-I during hibernation [15] contributes to cholesterol balance by supporting HDL formation. In addition, cell proliferation in the intestinal epithelium, which is suppressed during torpor, resumes during IBA periods [53] which would require a readily-available pool of fatty acids and cholesterol to support membrane formation.

Our results also revealed several differences in lipid handling between active and hibernating squirrels. Lipid-derived ketones serve as an energy source for brain and heart during arousal from torpor [54] and the importance of this metabolic fuel is underscored by the upregulation of the BHB transporter, MCT-1 in brain and increased expression of succinyl CoA transferase, the rate-limiting enzyme for ketolysis, in heart [55]. Levels of circulating BHB reported here, and of non-esterified free fatty acids (NEFAs) reported previously [56] are relatively low during normothermic states (SPR, SUM, IBA) compared with high levels during torpor, a pattern that has been observed in other studies [57], [58], [59], [60]. In contrast, plasma TGs were higher during IBA than LT in hibernators. Liver TGs tended to be lower in IBA compared with EN and LT hibernators, and a similar trend occurred for liver FFAs. These findings suggest that TGs and FFAs are released from the liver into the plasma during arousal from torpor or early in IBA periods to provide fuel during these energetically demanding times. Conversely, ketones and NEFAs are important fuel sources during torpor bouts.

Hibernating species must accumulate sufficient TG reserves during the active season to support energetic demands in winter. MGAT catalyzes the addition of a fatty acid acyl-CoA to monoacylglycerol to form diacylglycerol, a key step in TG synthesis. We hypothesized that MGAT activity would be elevated during the active season to promote TG synthesis. However, there were no differences in liver MGAT activity among SPR, LT and IBA animals. Previous studies on seasonal MGAT activity in hibernating species yielded conflicting results. Liver MGAT activity was higher in summer than in torpid golden mantled ground squirrels [61], but did not change seasonally in marmots [62].

Dramatic changes in nutrition, metabolism and fuel utilization are hallmarks of the hibernation phenotype, and make ground squirrels and other hibernators valuable models for exploring the capacity of mammals to tolerate extreme physiological states. Our study identified some of the pathways associated with altered cholesterol and lipoprotein profiles during the extended winter fast. How physiological systems safely manage large fluxes of lipids and sterols during the annual hibernation cycle is an exciting question that may lead to new avenues for therapeutic management of lipid disorders in humans.

## Supporting Information

Table S1 **Primer sequences of genes examined in this study.** Abbreviations: ABCA1, ATP-binding cassette transporter 1, ABCG8, ATP-binding cassette sub-family G member 8, ApoA-I, apolipoproteinA-I, ApoA-IV, apolipoproteinA-IV, DGAT2, diglyceride acyltransferase 2, HMGR, HMG-CoA reductase, LDLR, low density lipoprotein receptor, LXR, liver x receptor, MGAT2, monoacylglycerol acyltransferase 2, MTTP, microsomal triglyceride transfer protein, NPC1L1, Neimann-Pick C1 like 1, SERBP1c and 2, and sterol regulatory element binding protein 1c and 2.
(DOCX)

Table S2 **Body masses (in g) of squirrels used for the following analyses:** plasma total cholesterol and TGs (Figs. 2A, C, E and 5A); lipoprotein cholesterol (Figs. 2D-F) and TGs (Fig. 5B); whole body and individual organ cholesterol (Table 1); Tissue cholesterol esters (CE), free cholesterol (FC) (Fig. 3), triglycerides (TGs), and free fatty acids (FFAs) (Figs. 5C-H); biliary lipids (Table 2); monoacylglycerol acyltransferase (MGAT) activity; plasma β-hydroxybutyrate (BHB) (Fig. 6). SPR, spring, SUM, summer, EN, entering torpor (T_b_ 20–25°C), ET, early torpor (1 day in torpor, T_b_ ∼5°C), LT, late torpor (>1 week in torpor, T_b_ ∼5°C), AR, arousing from torpor (T_b_ 20–25°C), IBA, interbout arousal (T_b_ ∼37°C). Values are means ± s.e.m. with sample sizes in parentheses. Values with the same letter, in the same row are not different. na, not available.
(DOCX)

Table S3 **Days spring (SPR) animals spent in warm room after terminating hibernation, summer (SUM) squirrels spent in captivity before use, and hibernating squirrels spend in the cold room before use in analyses.** Hibernator activity states were: EN, entering torpor (T_b_ 20–25°C), ET, early torpor (1 day in torpor, T_b_ ∼5°C), LT, late torpor (>1 week in torpor, T_b_ ∼5°C), AR, arousing from torpor (T_b_ 20–25°C), and IBA, interbout arousal (T_b_ ∼37°C). Analyses included plasma total cholesterol and TGs (Figs. 2A, C, E and 5A); lipoprotein cholesterol (Figs. 2D–F) and TGs (Fig. 5B); whole body and individual organ cholesterol (Table 1); Tissue cholesterol esters (CE), free cholesterol (FC) (Fig. 3), triglycerides (TGs), and free fatty acids (FFAs) (Figs. 5C–H); biliary lipids (Table 2); monoacylglycerol acyltransferase (MGAT) activity; plasma β-hydroxybutyrate (BHB) (Fig. 6). Values are means ± s.e.m. with ranges in parentheses. See Table S2 for sample sizes. na, not available.
(DOCX)
