# Supplementary material for: Cholesterol and Lipoprotein Dynamics in a Hibernating Mammal
Source: PLoS One. 2011 Dec 15;6(12):e29111. doi: 10.1371/journal.pone.0029111 (PMC3240636; doi:10.1371/journal.pone.0029111)
Supplement: Table S1 — Primer sequences of genes examined in this study. Abbreviations: ABCA1, ATP-binding cassette transporter 1, ABCG8, ATP-binding cassette sub-family G member 8, ApoA-I, apolipoproteinA-I, ApoA-IV, apolipoproteinA-IV, DGAT2, diglyceride acyltransferase 2, HMGR, HMG-CoA reductase, LDLR, low density lipoprotein receptor, LXR, liver x receptor, MGAT2, monoacylglycerol acyltransferase 2, MTTP, microsomal triglyceride transfer protein, NPC1L1, Neimann-Pick C1 like 1, SERBP1c and 2, and sterol regulatory element binding protein 1c and 2. (DOCX) [file pone.0029111.s001.docx]

| **Gene** | **Forward Sequence** | **Reverse Sequence** |
| --- | --- | --- |
| **ABCA1** | GAA GGT TGC CAC AGC TTC TC | CAT GGT CTT GGC CAG GTA |
| **ACAT** | TTC GTT CTT TGC CTT TTT GCA | CAT CCT GTC ACC AAA GCG TAA |
| **ABCG8** | CAC CTT CCA CAT GTC CTC CT | GAC TTC AGG TCC ATG GCA GT |
| **ApoA-I** | CAC AGT GGC GAA ATC CTT CA | CCA CAC CCT GCA GGA TGA AA |
| **ApoA-IV** | AGC CAG ATG TTC GGG GAC AAC | CTC CTC AGC GTT CTT CTT CAT |
| **DGAT2** | AGC CCC CAG TCA GAG GAG AAG | GGA GGC CAC CGA AGT TAG CAA GAA |
| **HMGR** | ACC GTG GGT GGT GGG AC | GCC CCT TGA ACA CCT AGC ATC |
| **LDLR** | AGT TCA TCC GAG CCA TTT TCA | AGC CGA TGC ATT CCT GAC TC |
| **LXRα** | GCT CTG CTC ATT GCC ATC AG | TGT TGC AGC CTC TCT ACT TGG |
| **MGAT2** | AGG GGC TCC GAG TCC AGC AG | CCC GGC CCT GGA GAC CAT GA |
| **MTTP** | TTC ATT CAG CAC CTC CGC AC | CAA AAA GTC CAG GAT GGC TCC C |
| **NPC1L1** | ATC CTC ATC CTG GGC TTT GC | GCA AGG TGA TCA GGA GGT TGA |
| **SREBP1c** | GGA GCC ATG GAT TGC ACA TT | CCT CTC TCA CCC CCA GCA TA |
| **SREBP2** | CTG CAG CCT CAA GTG CAA AG | CAG TGT GCC ATT GGC TGT CT |
