# Supplementary material for: Cholesterol and Lipoprotein Dynamics in a Hibernating Mammal
Source: PLoS One. 2011 Dec 15;6(12):e29111. doi: 10.1371/journal.pone.0029111 (PMC3240636; doi:10.1371/journal.pone.0029111)
Supplement: Table S2 — Body masses (in g) of squirrels used for the following analyses: plasma total cholesterol and TGs (Figs. 2A, C, E and 5A); lipoprotein cholesterol (Figs. 2D-F) and TGs (Fig. 5B); whole body and individual organ cholesterol (Table 1); Tissue cholesterol esters (CE), free cholesterol (FC) (Fig. 3), triglycerides (TGs), and free fatty acids (FFAs) (Figs. 5C-H); biliary lipids (Table 2); monoacylglycerol acyltransferase (MGAT) activity; plasma β-hydroxybutyrate (BHB) (Fig. 6). SPR, spring, SUM, summer, EN, entering torpor (Tb 20–25°C), ET, early torpor (1 day in torpor, Tb ∼5°C), LT, late torpor (>1 week in torpor, Tb ∼5°C), AR, arousing from torpor (Tb 20–25°C), IBA, interbout arousal (Tb ∼37°C). Values are means ± s.e.m. with sample sizes in parentheses. Values with the same letter, in the same row are not different. na, not available. (DOCX) [file pone.0029111.s002.docx]

|  | **SPR Fast** | **SPR Fed** | **SUM Fast** | **SUM Fed** | **EN** | **ET** | **LT** | **AR** | **IBA** |
| --- | --- | --- | --- | --- | --- | --- | --- | --- | --- |
| **Plasma Cholesterol and TG** | 153.0 ± 10.4 (6) | 161.0 ± 10.4  (6) | 169.2 ± 19.9  (6) | 160.1 ± 7.6  (6) | na | na | 147.4 ± 11.6  (6) | na | 141.0 ± 8.1  (6) |
| **Lipoprotein Cholesterol and TG** | 153. ± 10.4^a,b^  (5) | na | 170.2 ± 10.2^a^  (6) | 173.8 ± 13.2^a^  (5) | na | na | 159.2 ± 13.0^a^  (5) | na | 127.5 ± 7.7 ^b^  (6) |
| **Whole Body Cholesterol** | na | 144.7 ± 9.0^a^  (3) | na | 162.3 ± 5.9^a^  (3) | na | na | 123.0 ± 5.9^b^  (6) | na | na |
| **Liver CE, FC, TG, FFA** | 159.8 ± 10.0^a,b^  (5) | na | 181.7 ± 11.9^a^  (6) | na | 144.1 ± 8.4^b,c^  (5) | na | 146.3 ± 11.3^b,c^  (6) | na | 130.8 ± 9.3^c^  (6) |
| **Intestinal CE, FC, TG, FFA** | 169.4 ± 3.6^a,b^  (5) | na | 181.7 ± 11.9^a^  (5) | na | 134.6 ± 10.2^c^  (4) | na | 126.3 ± 12.1^c^  (6) | na | 148.8 ± 8.3^b,c^  (6) |
| **WAT CE, FC, TG, FFA** | 157.2 ± 2.9  (6) | na | 181.7 ± 11.9  (5) | na | 135.2 ± 14.7  (5) | na | 154.5 ± 2.6  (6) | na | 143.4 ± 13.1  (5) |
| **Biliary Lipids** | na | na | na | 197.7 ± 7.9^a^  (7) | na | na | 119.7 ± 4.2^b^  (6) | na | na |
| **MGAT Activity** | 159.8 ± 10.0  (5) | 186.8 ±16.2  (5) | na | na | na | na | 145.2 ± 12.3  (5) | na | 135.4 ± 8.6  (5) |
| **Plasma BHB** | na | na | 155.7 ± 6.4  (5) | 174.2 ±11.8  (5) | na | 142.0 ± 13.7  (4) | 146.8 ± 15.4  (5) | 126.5 ± 4.7  (4) | 143.2 ± 7.7  (6) |
