# Supplementary material for: Cholesterol and Lipoprotein Dynamics in a Hibernating Mammal
Source: PLoS One. 2011 Dec 15;6(12):e29111. doi: 10.1371/journal.pone.0029111 (PMC3240636; doi:10.1371/journal.pone.0029111)
Supplement: Table S3 — Days spring (SPR) animals spent in warm room after terminating hibernation, summer (SUM) squirrels spent in captivity before use, and hibernating squirrels spend in the cold room before use in analyses. Hibernator activity states were: EN, entering torpor (Tb 20–25°C), ET, early torpor (1 day in torpor, Tb ∼5°C), LT, late torpor (>1 week in torpor, Tb ∼5°C), AR, arousing from torpor (Tb 20–25°C), and IBA, interbout arousal (Tb ∼37°C). Analyses included plasma total cholesterol and TGs (Figs. 2A, C, E and 5A); lipoprotein cholesterol (Figs. 2D–F) and TGs (Fig. 5B); whole body and individual organ cholesterol (Table 1); Tissue cholesterol esters (CE), free cholesterol (FC) (Fig. 3), triglycerides (TGs), and free fatty acids (FFAs) (Figs. 5C–H); biliary lipids (Table 2); monoacylglycerol acyltransferase (MGAT) activity; plasma β-hydroxybutyrate (BHB) (Fig. 6). Values are means ± s.e.m. with ranges in parentheses. See Table S2 for sample sizes. na, not available. (DOCX) [file pone.0029111.s003.docx]

|  | **SPR Fast**  **Time in Warm Room** | **SPR Fed**  **Time in Warm Room** | **SUM Fast**  **Time in Captivity** | **SUM Fed**  **Time in Captivity** | **EN**  **Time in Hibernation** | **ET**  **Time in Hibernation** | **LT**  **Time in Hibernation** | **AR**  **Time in Hibernation** | **IBA**  **Time in Hibernation** |
| --- | --- | --- | --- | --- | --- | --- | --- | --- | --- |
| **Plasma Cholesterol and TG** | 35.6 ± 0.3  (35-36) | 35.8 ± 0.2  (35-36) | 57.8 ± 13.0  (24-82) | 39.8 ± 11.3  (27-85) | na | na | 70.1 ± 9.8  (25-102) | na | 84.8 ± 12.5  (47-124) |
| **Lipoprotein Cholesterol and TG** | 35.5 ± 0.3  (35-36) | na | 39.3 ± 14.8  (20-103) | 42.2 15.9  (21-102) | na | na | 99.2 ± 6.1  (87-118) | na | 89.2 ± 11.3  (53-124) |
| **Whole Body Cholesterol** | na | 31.0 ± 0.0  (31 for all) | na | 40.0 ± 0.0  (40 for all) | na | na | 66.7 ±3.2  (53-77) | na | na |
| **Liver CE, FC, TG, FFA** | 35.8 ± 2.5  (33-44) | na | 28.0 ± 0.0  (28 for all) | na | 63.4 ± 11.5  (29-99) | na | 72.2 ± 5.5  (48-118) | na | 76.6 ± 4.5  49-129) |
| **Intestinal CE, FC, TG, FFA** | 52.4 ± 2.7  (50-62) | na | 28.0 ± 0.0  (28 for all) | na | 45.3 ± 10.3  (28-74) | na | 84.8 ± 14.1  (38-125) | na | 67.3 ± 4.5  49-129) |
| **WAT CE, FC, TG, FFA** | 36.8 ± 2.5  (33-44) | na | 28.0 ± 0.0  (28 for all) | na | 63.0 ± 11.4  (29-99) | na | 58.2 ± 8.2  (31-80) | na | 51.4 ± 8.6  (33-84) |
| **Biliary Lipids** | na | na | na | 53.7 ± 7.8  (29-66) | na | na | 69.7 ± 7.2  (60 - 105) | na | na |
| **MGAT Activity** | 35.8 ± 2.5  (33-44) | na | na | na | na | na | 77.0 ± 11.3  (56-118) | na | 73.4 ± 13.4  (49-124) |
| **Plasma BHB** | na | na | 53.5 ± 12.1  (28-86) | 48.8 ± 11.5  (27-81) | na | 74.0 ± 9.3  (57-89) | 73.5 ± 11.4  (25-102) | 71.5 ± 14.7  (44-97) | 74.3 ± 11.4  (47-124) |
